# Death from an Overdose of Nitrate of Potass

**Published:** 1845-01

**Authors:** 


*Death from an Overdose of Nitrate of Potass.*—An inquest has been lately held at Manchester, before Mr. Hudson, the Coroner, on the body of Wm. Ellison, aged about 60, who met his death under the following circumstances:

The deceased came originally from Runcorn, but had resided in the vicinity where he died, a number of years. He was in very indigent circumstances, getting his living by labourer's work. He had a sister and cousin residing in the town, by whom he was occasionally visited, but was generally regarded as a man of taciturn, reserved disposition, and held little communication with any body. For some time he had been troubled with scurvy, which affected his eyes, and he at length determined to get something to cure that disorder. He got a three-gill bottle from his cousin, three parts filled with water, and went with it to a druggist, whose assistant, at the request of the deceased, put into it one ounce of sulphur, two ounces of saltpetre, and half an ounce of cream of tartar. All these were put into the bottle, and mixed up together, the different witnesses understanding from the deceased, that the mixture was to stand in solution for three days, and then a portion of it might be taken; but none of them knew how much.

This mixture had been recommended by a woman, who had found it efficacious in a similar case. On one of the following mornings the deceased was observed by the neighbours, residing in the same yard, to go to the necessary several times; indeed, he was described as constantly running to and from that place; but no one appeared to take particular notice, not liking to interfere in his affairs. At half past eleven o'clock, a groan was heard proceeding from the necessary; but the deceased came out again, apparently very ill. At one o'clock he was observed to re-enter, and not coming out for half an hour, the neighbours began to confer with each other, and on one of them going in to see what he was doing, the deceased was found crouched down in one corner quite dead. It was proved in evidence, that up to that morning he had been in perfect health, and had been following his ordinary occupation; that he was, in fact, a hale old man, and with the exception of a slight touch of scurvy, was not subject to any disorder which would at all account for his sudden demise; the attention of the jury was, therefore, very naturally directed to the composition of the mixture which he was said to have taken, and to the effects which the ingredients of that compound would be likely to have upon his system. The remainder of the mixture, not more than half a gill, was introduced, and Mr. John Raynor, surgeon, sent for, and all the previous facts of the case having been recited to him by the coroner, Mr. Raynor at once concluded that the death of the deceased had been caused by an overdose of saltpetre, which had produced inflammation of the mucous membrane of the stomach and bowels, under which the deceased had sunk.

The quantity of saltpetre swallowed by the poor man was calculated at about ten drachms, and the jury at once returned a verdict: "Died from an overdose of saltpetre incautiously taken." The druggist's apprentice was reprimanded by the coroner for selling the mixture without giving information as to the properties of its ingredients.—*Dublin Journal.*

